# Biomarkers of Calcification, Endothelial Injury, and Platelet-Endothelial Interaction in Patients with Aortic Valve Stenosis

**DOI:** 10.3390/ijms26104873

**Published:** 2025-05-19

**Authors:** Paweł Bańka, Klaudia Męcka, Adrianna Berger-Kucza, Karolina Wrona-Kolasa, Anna Rybicka-Musialik, Beata Nowak, Marek Elżbieciak, Magdalena Mizia-Szubryt, Wojciech Wróbel, Tomasz Francuz, Michał Lelek, Agnieszka Kosowska, Wojciech Garczorz, Tomasz Bochenek, Andrzej Swinarew, Jarosław Paluch, Maciej Wybraniec, Katarzyna Mizia-Stec

**Affiliations:** 1 First Department of Cardiology, School of Medicine in Katowice, Medical University of Silesia, 40-635 Katowice, Poland; 2 Centre of the European Reference Network for Rare, Low Prevalence or Complex Diseases of the Heart (ERN GUARD Heart), 40-635 Katowice, Poland; 3 Department of Biochemistry, Faculty of Medical Sciences in Katowice, Medical University of Silesia, 40-752 Katowice, Poland; 4 Faculty of Science and Technology, University of Silesia, 41-500 Chorzów, Poland; 5 Institute of Sport Science, The Jerzy Kukuczka Academy of Physical Education, 40-065 Katowice, Poland; 6 Institute of Genetics and Animal Biotechnology of the Polish Academy of Sciences, 05-552 Jastrzębiec, Poland; 7 Department of Laryngology, Faculty of Medical Sciences in Katowice, Medical University of Silesia, 40-055 Katowice, Poland

**Keywords:** aortic stenosis, biomarkers, autotaxin, lysophosphatidic acid, growth differentiation factor-15, thrombomodulin, flow-mediated dilatation, endothelial injury, valvular interstitial cells, platelets

## Abstract

Aortic stenosis (AS) is a progressive valvular heart disease characterized by fibrocalcific remodeling, inflammation, and hemodynamic disturbances. Serum biomarkers may indirectly reflect these processes. Autotaxin (ATX) and lysophosphatidic acid (LPA) have been implicated in osteogenic differentiation of valvular interstitial cells, while growth differentiation factor-15 (GDF-15) reflects cellular stress and vascular changes. Thrombomodulin (TM) indicates endothelial injury and interacts with thrombin. This study aimed to evaluate biomarkers focusing on serum ATX, LPA, GDF-15, and TM levels and flow-mediated dilatation (FMD) in patients with AS. Overall, 149 patients were included in the study: 86 consecutive patients with AS hospitalized due to qualification for invasive treatment of AS and 63 controls. The clinical characteristics, echocardiographic data, FMD, and the following biomarkers—ATX, LPA, GDF-15, and TM—were included in the analysis. AS patients presented increased serum levels of ATX, GDF-15, and TM as compared to the controls. Differences in LPA levels were not statistically significant. FMD values were significantly lower in AS patients. The biomarkers mentioned above and FMD correlated with AS severity. There were no differences in both biomarkers’ serum levels and FMD regarding the hemodynamic AS phenotype. GDF-15 serum level was a risk factor for all-cause mortality and MACCE in the 12-month follow-up.

## 1. Introduction

Aortic stenosis (AS) is one of the most common valvular heart diseases in the elderly and presents a significant clinical challenge due to its progressive nature and high mortality rate in advanced stages [1,2]. Inflammatory processes, tissue remodeling, and hemodynamic disturbances play a crucial role in the pathophysiology of this disease [3]. Recent studies indicate that biochemical markers such as autotaxin (ATX), lysophosphatidic acid (LPA), growth differentiation factor-15 (GDF-15), and thrombomodulin (TM) may significantly contribute to the development and progression of AS [4,5,6]. ATX is an enzyme associated with lipid metabolism and has been shown to influence inflammatory pathways, while GDF-15 plays a role in cellular stress processes and may influence vascular changes. TM is a membrane-bound glycoprotein found primarily on the surface of endothelial cells, where it serves as a crucial modulator of blood coagulation. Beyond its anticoagulant function, TM also exhibits anti-inflammatory and protective properties, contributing to the maintenance of vascular stability. AS is a progressive, chronic disease that spans a spectrum beginning with mild fibrocalcific leaflet changes, termed aortic sclerosis, and progressing to more severe calcification with end-stage significant obstruction of left ventricular ejection. The aortic valve leaflets can gradually progress to valvular sclerosis, and the pathological mechanism is thought to be similar to that of atherosclerosis, including valve endothelial damage, lipid deposition, and inflammatory response [7]. The advanced stage is characterized by the presence of extracellular fibrosis and calcification. Aberrant differentiation of valvular interstitial cells (VICs) into osteoblasts may be the theoretical basis for ectopic ossification of the aortic valve [8]. Recent evidence indicates that ATX and lysophosphatidic acid (LPA) promote an osteogenic program in VICs [9,10]. It is postulated that activated blood platelets, which induce the release of ATX from VICs, may play a crucial role in the pathogenesis of AS. In physiological conditions, circulating platelets do not react with the vascular endothelium. Damage to the endothelium immediately induces platelet adhesion and aggregation. In addition, activated platelets have the ability to modulate endothelial cell properties. The interaction between platelets and the endothelium is a critical factor in thrombogenesis. TM expression has been demonstrated to influence platelet function [11]. Endothelial cells deficient in TM exhibit increased platelet string formation under flow conditions, highlighting TMs role in modulating platelet-endothelium interactions [12]. All of the above markers may indirectly reflect the pathomechanism of processes that underlie the development and severity of AS [13,14]. Conversely, these markers could serve as targets for therapeutic interventions and as prognostic indicators. Thus, the aim of this study was to compare serum concentrations of biomarkers in patients with AS and a control group, to evaluate the biomarkers in relation to the severity and hemodynamic phenotype of AS, and to verify their prognostic role in the AS population.

## 2. Results

### 2.1. Clinical Characteristics

A comparative analysis of clinical and biochemical parameters revealed significant differences between patients with AS and the control group. Patients with AS exhibited a lower prevalence of hyperlipidemia (88% vs. 98.4%, *p* = 0.018), current smoking (15.7% vs. 42.9%, *p* = 0.001), coronary artery disease (68.2% vs. 85.7%, *p* = 0.027), lower CCS (Canadian Cardiovascular Society) class (1.2 ± 1.1 vs. 2.2 ± 1.4, *p* < 0.001), and higher NYHA (New York Heart Association) class (2.5 ± 0.8 vs. 1.5 ± 1.1, *p* < 0.001). The clinical characteristics are shown in Table 1 and the laboratory characteristics are shown in Table S1 (Supplementary Materials).

### 2.2. Echocardiographic Characteristics of AS Group

The echocardiographic characteristics of the study group were as follows: aortic valve area (AVA) 0.8 ± 0.2 cm^2^, maximum velocity (Vmax) 4.2 ± 0.7 m/s, maximum pressure gradient (Pmax) 74 ± 23.1 mmHg, and mean pressure gradient (Pmean) 44.1 ± 14.8 mmHg. Among the study group, 77 patients were diagnosed with severe AS (39 females, 38 males, mean age: 75.95 ± 10.5 years), while 9 patients were diagnosed with moderate AS (3 females, 6 males, mean age: 65.11 ± 20.6 years).

The analysis of Pmean, stroke volume index (SVi), and left ventricular ejection fraction (LVEF) revealed the following hemodynamic subgroups of patients with severe AS: 53 high-gradient AS (24 females, 29 males, mean age: 76.28 ± 11.2 years), 15 low-flow low-gradient AS with preserved LVEF (9 females, 6 males, mean age: 77.6 ± 6.7 years), and 2 low-flow low-gradient AS with reduced LVEF (2 females, 0 males, mean age: 84 ± 1.4 years).

A comparative analysis of echocardiographic parameters between the study groups revealed several differences that corresponded to the baseline pathology. Specifically, the study observed increased thickness of the interventricular septum (IVS) (14.7 ± 3.2 vs. 11.9 ± 2.5 mm, *p* < 0.001) and posterior wall (PW) (11.1 ± 2.0 mm vs. 9.6 ± 1.5 mm, *p* < 0.001) in AS as compared to the control group. The LV end-diastolic diameter (EDD) was significantly smaller in the AS group (47.8 ± 6.1 mm) compared to the control group (50.5 ± 7.1 mm, *p* = 0.018). LV end-systolic volume (ESV) was also lower in the AS group (52.5 ± 31 mL) than in the control group (55 ± 18.2 mL, *p* = 0.036). LVEF was slightly higher in the AS group (54.6 ± 10.8%) compared to the control group (52.6 ± 7.7%, *p* = 0.023). LV global longitudinal strain (GLS) was significantly reduced in the AS group (−14 ± 3.1%) compared to the control group (−15.7 ± 3.7%, *p* = 0.015).

Valvulo-arterial impedance (Zva) was significantly increased in the AS group (5.6 ± 1.7 mmHg/mL/m^2^) compared to the controls (4.8 ± 1.4 mmHg/mL/m^2^, *p* = 0.003). The left atrial (LA) area was significantly larger in the AS group (24.1 ± 6.2 cm^2^) than in the control group (21.3 ± 4.9 cm^2^, *p* = 0.009). Finally, the ratio of early mitral inflow velocity to early diastolic mitral annular velocity (E/E ratio) was significantly higher in the AS group (15.6 ± 7) compared to the control group (10 ± 3.9, *p* < 0.001). The echocardiography characteristics are shown in Table 2.

### 2.3. Flow-Mediated Dilation

The results demonstrated that FMD was significantly lower in the AS group (5.8 ± 4.7% vs. 9.9 ± 6.5%; *p* < 0.001). FMD exhibited a moderate positive correlation with AVA (r = 0.327, *p* < 0.001) and a moderate negative correlation with both V max (r = −0.310, *p* < 0.001) and *p* mean (r = −0.327, *p* < 0.001).

### 2.4. Biomarker Serum Levels: AS vs. Controls

The serum levels of the biomarkers exhibited the following differences: a significant increase in ATX (112.07 ± 50.0 vs. 93.54 ± 27.2 ng/mL, *p* = 0.003), GDF-15 (1.34 ± 1.4; Q1: 0.64; Q3: 1.5 vs. 0.71 ± 0.5; Q1: 0.32; Q3: 0.94 ng/mL, *p* < 0.001), and TM (7.76 ± 4.0 vs. 6.3 ± 1.9 ng/mL, *p* = 0.005) were observed in the AS as compared to the control group. There were no significant differences in the LPA concentrations between the groups. The results are shown in Table 3.

### 2.5. Biomarker Serum Levels: Hemodynamic Subtypes of Patients with Severe AS

No significant differences were observed in the serum levels of biomarkers or in FMD between the hemodynamic subtypes of patients with severe AS: high gradient severe AS vs. low-flow low-gradient severe AS. The results are shown in Table 4. A comparative analysis was conducted between the high-gradient AS and the control group, as well as between the low-gradient AS and the control group. The biomarkers ATX, TM, and GDF-15 exhibited significantly higher levels in both high-gradient AS and low-gradient AS compared to the control group, consistent with prior findings.

### 2.6. Correlations and Regression Analysis: Serum Biomarkers, FMD, and Parameters of AS Severity

The regression analysis revealed significant correlations between serum biomarkers ATX, GDF-15, and TM and parameters of AS severity. Notably, GDF-15 presented the strongest relationships across these variables. Serum GDF-15 levels correlated with AVA (r = −0.410, *p* < 0.001), Vmax (r = 0.374, *p* < 0.001), and Pmean (r = 0.362, *p* < 0.001). The results of Spearman’s correlation analysis are shown in Table 5.

FMD also correlated with the parameters of AS severity: AVA (r = 0.327, *p* = 0.001), Vmax (r = −0.310, *p* = 0.001), and Pmean (r = −0.327, *p* = 0.001). In order to clarify the degree of relationship between the variables, regression analysis was conducted, which confirmed the more important relationships. The results of regression analyses together with the R² values are shown in Table 6.

### 2.7. 12-Month Follow-Up

During the 12-month follow-up period, 22 (25.6%) patients with AS underwent surgical aortic valve replacement, 35 (40.7%) patients underwent transcatheter aortic valve implantation, 13 (15.1%) patients declined the intervention, 9 (10.5%) patients qualified for conservative treatment due to moderate AS, and 7 (8.1%) patients qualified for conservative treatment due to severe comorbidities.

The mortality rate was found to be significantly higher in patients with AS, with 14 (16.3%) deaths observed compared to 1 (1.6%) death in the control group (*p* = 0.003). Among patients with AS, death was recorded in 10 (34.5%) patients treated conservatively compared to 1 (4.5%) patient undergoing surgical aortic valve replacement and 3 (8.6%) patients undergoing transcatheter aortic valve replacement (*p* = 0.001).

Major adverse cardiovascular and cerebrovascular events (MACCE) were reported in 18 (20.9%) AS patients compared to 8 (12.7%) controls (*p* = 0.191). Among patients with AS, MACCE were observed more frequently in patients treated conservatively, 11 (37.9%) compared to those undergoing surgical aortic valve replacement, 2 (9.1%), and transcatheter aortic valve replacement, 5 (14.3%) (*p* = 0.015). The incidence of major adverse cardiovascular events (MACE), bleeding, and mortality during follow-up is demonstrated in Table S2 (Supplementary Materials).

### 2.8. Risk Factors for Total Mortality and MACCE

#### 2.8.1. Mortality

The analysis revealed the following mortality risk factors in the AS group: age (OR = 1.0897, 95% CI: 1.0183–1.1662, *p* = 0.013), atrial fibrillation (OR = 4.4815, 95% CI: 1.0286–19.5246, *p* = 0.046), and NYHA class (OR = 2.6235, 95% CI: 1.2154–5.6629, *p* = 0.014). Additionally, reduced estimated glomerular filtration rate (eGFR) (OR = 0.9716, 95% CI: 0.9453–0.9986, *p* = 0.04) and increased right ventricular systolic pressure (RVSP) (OR = 1.0055, 95% CI: 1.003–1.1097, *p* = 0.038) were associated with mortality risk. Among the analyzed biomarkers, GDF15 serum level (OR = 1.0006, 95% CI: 1.0000–1.0011, *p* = 0.036) was associated with increased mortality risk.

#### 2.8.2. MACCE

The analysis identified several significant risk factors for MACCE: age (OR = 1.05, 95% CI: 1.01–1.10, *p* = 0.029), atherosclerotic plaque in the ascending aorta (OR = 8.45, 95% CI: 1.91–37.35, *p* = 0.0049), and eGFR (OR = 0.963, 95% CI: 0.9414–0.9851, *p* = 0.0011). Among the biomarkers that were analyzed, only the serum level of GDF15 (OR = 1.0006, 95% CI: 1.0002–1.0011, *p* = 0.001) was associated with an increased risk of MACCE. The predictive factors for mortality and MACCE are summarized in Table 7.

### 2.9. Main Findings

The study demonstrated that patients with AS exhibited distinct clinical, echocardiographic, and biochemical profiles compared to controls. Notably, serum levels of ATX, GDF-15, and TM were significantly elevated in AS patients, while LPA levels showed no difference. Impaired endothelial function, as indicated by reduced FMD, was observed in the AS group and correlated with AS severity. Echocardiographic analysis revealed that AS patients had reduced LV GLS and increased Zva. Among biomarkers, GDF-15 showed the strongest association with disease severity and independently predicted both mortality and MACCE. While ATX was elevated in AS, it did not vary significantly across hemodynamic subtypes. The 12-month follow-up confirmed a higher mortality and MACCE rate among conservatively treated patients.

## 3. Discussion

The present study demonstrates the serum levels of biomarkers, including ATX, LPA, GDF-15, and TM levels, as well as FMD, which serves as a surrogate index of endothelial function [15], in patients with AS. We have analyzed all markers in relation to the severity and hemodynamic phenotype of AS and verified their prognostic role in this population.

Our results show significant differences in biomarkers’ levels between AS patients and controls as well as significant correlations between ATX, GDF-15, TM levels, and parameters of AS severity, providing insights into the underlying pathophysiological mechanisms and their potential clinical implications.

The results suggest the role of inflammatory and endothelial dysfunction in the progression of AS. In particular, ATX and GDF-15 levels were significantly higher in AS patients than in controls.

ATX, an enzyme involved in lipid metabolism and inflammatory pathways, is implicated in vascular calcification and fibrosis [16]. ATX is responsible for converting lysophosphatidylcholine into LPA, a bioactive lipid mediator in numerous biological processes, including inflammation, fibrosis, and osteogenesis [17]. The observed increase in ATX levels suggests its role in AS pathogenesis, probably through modulation of valvular interstitial cells (VICs) and promotion of osteogenic differentiation.

The ATX-LPA pathway may be a novel therapeutic target, as its inhibition may slow down fibrosis and inflammation [16].

This result aligns with previous research suggesting that ATX plays a key role in the inflammatory and calcific processes underlying valvular degeneration [18,19,20]. ATX expression was notably increased in vascular endothelial cells, adipose tissue, and inflammatory environments [18]. Elevated levels of ATX have been associated with various cardiovascular diseases, including aortic stenosis, where it contributes to vascular calcification and fibrosis [19]. In addition, studies have indicated that aortic valve calcification is enhanced by ATX derived from lipoprotein (a) and valve interstitial cells, thereby accelerating the progression of the disease [20]. ATX has also been associated with atherosclerosis and endothelial dysfunction, further exacerbating cardiovascular conditions. Given its role in inflammation and fibrosis, ATX is considered a potential therapeutic target [21]. In the present study, ATX serum levels correlated with disease severity, suggesting its potential as a promising biomarker for risk assessment in patients with AS.

Interestingly, despite the clear elevation of ATX in the AS group, LPA levels did not differ significantly between the patients and controls. This finding requires careful interpretation. Several possible explanations may account for this apparent discrepancy. LPA is rapidly degraded in circulation by lipid phosphate phosphatases, making it difficult to detect meaningful changes in serum levels, even when its production is increased locally [22]. LPA acts primarily in a paracrine or autocrine manner, exerting its effects in the immediate microenvironment of the valve tissue rather than being systemically elevated [22,23]. This localized activity may not be reflected in peripheral blood measurements. The measurement method and stability of LPA in serum may also contribute to variability and underestimation, given its short half-life and susceptibility to preanalytical factors. Thus, while LPA is mechanistically central to ATXs effects, it may not serve as a reliable systemic biomarker for AS. In contrast, ATX, as a more stable circulating protein, appears to better reflect the ongoing pathological processes in the valve and vascular endothelium.

According to the literature data, LPA has been implicated in the activation of valve interstitial cells, which contributes to increased fibrosis and calcification of the aortic valve [23]. It also induces endothelial dysfunction by enhancing oxidative stress and inflammatory signaling pathways [24]. Elevated LPA levels have been detected in patients with atherosclerosis, thrombosis, and other vascular diseases [25]. Furthermore, LPA has been implicated in immune cell recruitment, which may contribute to inflammation and tissue damage in AS [26].

The study also demonstrated elevated levels of TM in patients with AS. It seems to be of importance because TM increase may reflect endothelial activation or damage, which are characteristic of advanced AS due to chronic hemodynamic stress. TMs role in modulating coagulation and inflammation further supports its relevance in the complex interplay between valvular pathology and systemic vascular responses. TM plays a crucial role in anticoagulation by binding to thrombin, converting it from a pro-coagulant to an activator of protein C, which in turn inhibits clot formation [27]. Beyond its anticoagulant properties, TM has been shown to possess anti-inflammatory and cytoprotective effects, which contribute to the maintenance of vascular homeostasis. In AS, TM levels may be altered due to endothelial dysfunction and chronic inflammation. TM levels have the potential to serve as a marker of endothelial integrity, and the preservation of endothelial function may contribute to a reduction in the risk of thrombotic complications in AS patients.

One of the key findings is the significantly higher serum concentration of GDF-15 in AS patients. GDF-15 is known to be involved in cellular stress responses, and its elevated levels likely reflect ongoing valvular and myocardial stress. The strong correlations observed between GDF-15 and echocardiographic parameters such as AVA, Vmax, and Pmean suggest that GDF-15 may be a sensitive marker of disease severity. Its inverse correlation with AVA and positive correlation with Vmax and Pmean indicate that GDF-15 increases as the valve becomes more stenotic. This may reflect both progressive inflammation and increasing mechanical strain on the myocardium. GDF-15, in particular, has emerged as a promising candidate for risk stratification, given its consistent correlation with AS severity parameters. A body of research has previously indicated that GDF-15 levels can serve as a predictor of adverse outcomes in cardiovascular diseases. Consequently, elevated GDF-15 levels could serve as a prognostic indicator in AS, aiding clinical decision-making processes. GDF-15, a stress-responsive cytokine, is a member of the transforming growth factor-beta (TGF-β) superfamily. It is expressed in multiple tissues, including the heart, liver, and endothelial cells, and is upregulated in response to inflammation, oxidative stress, and hypoxia. GDF-15 is increasingly recognized as an important biomarker in cardiovascular diseases, including heart failure, coronary artery disease, and aortic stenosis [28,29,30]. In patients with AS, elevated GDF-15 levels have been associated with increased myocardial stress, fibrosis, and worse clinical outcomes [5,31].

Notably, no statistically significant differences in biomarker levels were observed between the high-gradient and low-gradient AS subgroups. This suggests that the biochemical processes reflected by ATX, GDF-15, and TM are active across different clinical forms of AS. However, the lack of statistical significance may be attributable to the predominance of the high-gradient subgroup and the limited sample size of the other subgroups.

The analysis of FMD provided additional evidence for endothelial dysfunction in AS. FMD was significantly lower in AS patients and showed clear correlations with AVA, Vmax, and Pmean. It confirms that the degree of direct index of endothelial impairment is related to the stage of valvular stenosis. These findings are in line with the concept that AS is not merely a localized valvular pathology but part of a broader systemic process.

Furthermore, at 12-month follow-up, patients with AS had higher mortality and MACCEs compared to controls. Among patients with AS, mortality and MACCEs were higher in patients treated conservatively compared to those who underwent surgical aortic valve replacement or transcatheter aortic valve implantation. The elevated incidence of MACCEs in the conservative treatment group can be attributed to the higher mortality rate observed in this cohort, as MACCEs encompassed fatal cardiovascular events. These observations underscore the critical nature of AS as a progressive condition, underscoring the need for effective treatment to mitigate the risk of mortality and prevent cardiovascular complications.

The progression of AS can be driven by a complex interplay between endothelial dysfunction, inflammation, and pathological calcification of the aortic valve. This study lends further credence to the hypothesis that activated platelets and ATX may enhance aortic valve calcification by LPA. The hypothesis is based on the premise that activated platelets release various mediators, including adenosine diphosphate, which binds to P2Y1 receptors on VICs. This, in turn, stimulates the release of ATX, an enzyme that catalyzes the production of LPA. LPA, in turn, has been shown to promote VIC mineralization by increasing the expression of osteogenic markers.

## 4. Materials and Methods

### 4.1. Materials

Overall, 149 adult patients (66 females, 83 males, mean age: 74.1 ± 9.8 years) were included in the study: 86 consecutive patients with AS (42 females, 44 males, mean age: 74.8 ± 12.2 years) and 63 (24 males, 39 males, mean age: 73.1 ± 4.6 years) controls.

Patients with AS admitted to a tertiary cardiology center for invasive treatment of AS between July 2021 and December 2023 were recruited for the study. The study group was a population with moderate to severe AS. Subjects fulfilling the criteria for aortic valve replacement according to the current guidelines were qualified for the invasive treatment [32,33]. The control group consisted of gender- and age-matched patients without aortic valve disease.

Patients in both the study and control groups underwent a thorough screening process, and their previous discharge summaries were analyzed to identify the following exclusion criteria: congenital heart disease, cancer, autoimmune disease, infection, pregnancy, hematologic disorders including congenital coagulation disorders (thrombophilia, hemophilia A/B), infective endocarditis, chronic kidney disease stage IV–V (eGFR < 30 mL/min), chronic dialysis, liver dysfunction (any hepatic aminotransferase > upper reference limit), and lack of informed consent for the study.

The study was conducted in accordance with the Declaration of Helsinki and approved by the local Bioethics Committee. Prior to enrollment, written informed consent was obtained from all participants.

### 4.2. Clinical Assessment

All patients enrolled in the study underwent a comprehensive medical evaluation, which included the collection of data on NYHA class, CCS class, and the presence of comorbidities (atrial fibrillation, hypertension, coronary artery disease, diabetes, dyslipidemia, hypothyroidism, history of smoking, peripheral artery disease, chronic obstructive pulmonary disease), pharmacotherapy. A standard physical examination was performed. The clinical evaluation of the cardiovascular system included the following: measurement of weight, height, BMI, systolic and diastolic blood pressure, heart rate, ECG, transthoracic 2D echocardiography, and flow-mediated dilatation (FMD).

Laboratory tests were performed in both groups as part of the standard diagnostic procedure. The comprehensive laboratory panel included a range of hematological and biochemical parameters, such as erythrocyte count, hemoglobin, hematocrit, MCV, white blood cell count, neutrophils, lymphocytes, platelets, MPV, PDW, ALT, glucose, creatinine, eGFR (Cockcroft-Gault formula), sodium, potassium, TSH, triglycerides, total cholesterol, LDL, and HDL. These comprehensive blood tests were obtained to facilitate the enrollment process for the study participants.

### 4.3. Transthoracic Echocardiography

Transthoracic echocardiography was performed using a 2.5-MHz probe in 2D, M, and Doppler modes by a highly experienced examiner according to the recommendations of the European Association of Cardiovascular Imaging [34]. Offline 2D speckle tracking imaging was employed to calculate LV GLS.

The following parameters were assessed: AVA, Vmax, Pmax, Pmean, LV EDD, LV end-systolic diameter (ESD), LV end-diastolic volume (EDV), LV ESV, left ventricular outflow tract diameter (LVOT), LVEF, SVi, LA area, IVS thickness, PW thickness, early diastolic mitral inflow velocity (E wave), early diastolic mitral annular velocity (E′ wave), E/E′ ratio, Zva, atherosclerotic plaque in ascending aorta, and RVSP.

Based on the parameters AVA, Vmax, Pmean, LVEF, and SVi, the hemodynamic subtypes of severe AS were defined: (1) high-gradient (Pmean ≥ 40 mmHg), (2) low-flow low-gradient (Pmean < 40 mmHg, Svi ≤ 35 mL/m^2^) with preserved LVEF, and (3) low-flow low-gradient (Pmean < 40 mmHg, Svi ≤ 35 mL/m^2^) with reduced LVEF.

### 4.4. Flow-Mediated Dilation

FMD was obtained following the previously published method [35]. Continuous measurements of brachial artery velocity and diameter were performed by duplex ultrasound using a high-frequency ultrasound device (Canon Medical Systems Corporation, Otawara, Japan; VIVID 7 Dimension, GE Healthcare, Chicago, IL, USA) equipped with a high-frequency vascular probe (7–10 MHz), an internal electrocardiogram monitor, and vascular software (for two-dimensional imaging, color, and spectral Doppler). ECG-gated end-diastolic B-mode images were analyzed. All measurements were performed by an experienced physician in all subjects using the same examination protocol and techniques to reduce inter- and intra-observer variability. Brachial artery examinations were performed in the morning in a quiet, darkened, temperature-controlled room after a rest period of at least 10 min. The measurements were obtained in the supine position. After positioning the sphygmomanometer cuff proximally to the visualized vessel, the baseline arterial diameter was assessed 5–10 cm above the antecubital fossa before cuff inflation. The brachial artery diameter was defined as the average of multiple measurements. The cuff was then inflated for 3 min to achieve vessel occlusion (200 mmHg or 50 mmHg above actual systolic blood pressure). The mean values of brachial artery velocity and diameter were obtained between −50 and −60 s after cuff deflation. The proportional difference between the baseline artery and the dilated artery after reactive hyperemia was calculated and defined as FMD.

### 4.5. Laboratory Tests—Biomarkers

Blood samples (10 mL) were collected from the antecubital vein of all study participants (those in both the study and control groups). The serum was obtained, aliquoted, and frozen immediately at −80 °C in polypropylene tubes.

#### 4.5.1. Fluorescent Bead-Based Luminex Assays

Serum samples were analyzed in panels using a magnetic Luminex assay, following the manufacturer’s instructions. The serum was diluted as per the manufacturer’s recommendations and assayed immediately. Protein concentrations of ATX, GDF-15, and TM were measured using multiplex, bead-based (Luminex, Minneapolis, MN, USA) assays on a Bio-Plex 200 suspension array system, in accordance with the manufacturer’s protocols (R&D Systems, Minneapolis, MN, USA). Data acquisition was performed on a validated and calibrated Bio-Plex 200 system (Bio-Rad Laboratories, Watford, UK) and analyzed using Bio-Plex Manager 6.0 software (Bio-Rad Laboratories, Watford, UK). The detection target was set to 50 beads per region, with a low RP1 target for CAL2 calibration, and doublet discriminator (DD) gates recommended at 5000–25,000 for Bio-Plex. The median fluorescence intensity (MFI) was recorded and analyzed.

#### 4.5.2. ELISA Assay

A competitive inhibition enzyme immunoassay kit (Cloud-Clone Corp.; Houston, TX, USA) was used for quantitative analysis of LPA in serum. The analysis was conducted according to the manufacturer’s manual. Briefly, serum samples after defrosting were centrifuged (1000× *g*, 10 min., 4 °C). Next, 50 µL of serum, standards, and blank were added to the plate, then 50 µL of Detection Reagent A was added immediately and incubated for 1 h. The plate was washed 3 times with 350 µL of Wash Solution, and 100 µL of Detection Reagent B was added for 30 min. The Plate was washed again, and 90 µL of Substrate Solution was added for 15 min. Finally, 50 µL of Stop Solution was added, and the absorbance was measured immediately at 450 nm with a correction at 540 nm using an Infinite M200 microplate reader (TECAN, Männedorf, Switzerland).

### 4.6. 12-Month Follow-Up

Patients were followed up for 12 months as part of regular outpatient clinic visits or, in selected cases, via mobile phone on an outpatient basis. Patients with AS were followed up for eligibility for surgical aortic valve replacement, transcatheter aortic valve implantation, or conservative management. The occurrence of death and MACCE was evaluated, defined as a composite of cardiovascular death, non-fatal myocardial infarction, and non-fatal stroke.

### 4.7. Statistical Analysis

Statistical analysis was performed using Statistica software (version 13.3, StatSoft, Kraków, Poland). Quantitative variables were presented as mean ± standard deviation (SD) or median (values of 1st and 3rd quartiles), and qualitative parameters were expressed as numbers and percentages. The Shapiro–Wilk test was used to verify the type of distribution. For normally distributed variables, the student’s *t*-test for unpaired samples was used, while the Mann–Whitney U test was implemented for non-normally distributed parameters. The chi-squared test was used for qualitative variables. The relationship between biomarker concentrations and other factors was determined using Spearman’s rank correlation coefficient. Multiple regression analysis included all variables with *p* < 0.1 in the univariate regression model. Statistically significant results were defined as those with *p*-values less than 0.05.

## 5. Conclusions

Our study highlights the significance of ATX, GDF-15, and TM as potential biomarkers of AS severity and progression. Elevated levels of these markers reflect the underlying inflammatory and endothelial dysfunction processes contributing to valvular calcification and stenosis. This study provides valuable insights into the clinical and biochemical landscape of AS. The identified correlations between biomarkers, echocardiographic parameters, and AS severity highlight potential avenues for early disease detection and risk stratification. Future research should focus on validating these biomarkers in larger cohorts. A thorough understanding of these relationships may help to identify new therapeutic targets and improve diagnostic methods for patients with AS.

## Figures and Tables

**Table 1:** Baseline clinical characteristics. BMI—body mass index; NYHA—New York Heart Association; CCS—Canadian Cardiovascular Society; AF—atrial fibrillation; CAD—coronary artery disease; COPD—chronic obstructive pulmonary disease; PAD—peripheral artery disease; eGFR—estimated glomerular filtration rate; TSH—thyroid-stimulating hormone; LDL—low-density lipoprotein; HDL—high-density lipoprotein.

	Patients with AS (N = 86)	Control Group (N = 63)	*p* Value
**Male (%), Female (%)**	44 (51.2%), 42 (48.8%)	39 (61.9%), 24 (38.1%)	0.192
**Age (mean, SD)**	74.8 ± 12.2	73.1 ± 4.6	0.69
**Height (cm)**	165.8 ± 8.9	167.9 ±13.4	0.077
**BMI (kg/m^2^)**	27.7 ± 4.4	28.5 ± 4.5	0.337
**NYHA class (mean, SD)**	2.5 ± 0.8	1.5 ± 1.1	0.001
**CCS class (mean, SD)**	1.2 ± 1.1	2.2 ± 1.4	0.001
**AF (%)**	16 (18,6%)	1 (1,6%)	0.001
**Hypertension (%)**	76 (92.7%)	57 (90.5%)	0.633
**CAD (%)**	58 (68.2%)	54 (85.7%)	0.027
**Diabetes (%)**	18 (21.4%)	22 (34.9%)	0.069
**Dyslipidemia (%)**	73 (88%)	62 (98.4%)	0.018
**Hypothyroidism (%)**	15 (18.1%)	6 (9.5%)	0.145
**Smoking (%)**	13 (15.7%)	27 (42.9%)	0.001
**COPD (%)**	7 (8.5%)	7 (11.1%)	0.603
**PAD (%)**	26 (31.3%)	11 (17.5%)	0.056
**Creatinine [mg/dL]**	1.2 ± 1.0	0.9 ± 0.2	0.001
**Sodium [mmol/L]**	138.9 ± 3.2	139.4 ± 2.1	0.832
**Potassium [mmol/L]**	4,2 ± 0.4	4.1 ± 0.4	0.011
**eGFR [mL/min]**	63.5 ± 22.3	75.5 ± 16	0.001
**TSH [μIU/mL]**	1.9 ± 0.9	1.9 ± 1.5	0.234
**Triglycerides [mg/dL]**	97.5 ± 32.5	118 ± 48.5	0.032
**Total cholesterol [mg/dL]**	156.5 ± 39.5	150.9 ± 45	0.215
**LDL [mg/dL]**	82.2 ± 33	78.9 ± 39.4	0.357
**HDL [mg/dL]**	54.8 ± 17.9	48.5 ± 12.4	0.039

**Table 2:** Echocardiography characteristics. LV—left ventricular; EDD—end-diastolic diameter; ESD—end-systolic diameter; EDV—end-diastolic volume; ESV—end-systolic volume; EF—ejection fraction; SVi—stroke volume index; GLS—global longitudinal strain; IVS—interventricular septum thickness; PW—posterior wall thickness; Zva—valvulo-arterial impedance; LA area—left atrium area; E/E′—ratio of early mitral inflow velocity to early diastolic mitral annular velocity.

	Patients with AS (N = 86)	Control Group (N = 63)	*p* Value
**Left Ventricular Parameters**			
**LV EDD [mm]**	47.8 ± 6.1	50.5 ±7.1	0.018
**LV ESD [mm]**	30.6 ± 7.5	31.2 ± 6.6	0.32
**IVS [mm]**	14.7 ± 3.2	11.9 ± 2.5	0.001
**PW [mm]**	11.1 ± 2.0	9.6 ± 1.5	0.001
**LV EDV [mL]**	113.4 ± 37.3	116.4 ± 27	0.298
**LV ESV [mL]**	52.5 ± 31	55 ± 18.2	0.036
**LV EF [%]**	54.6 ± 10.8	52.6 ± 7.7	0.023
**LV SVi [mL/m^2^]**	35 ± 10.5	31.3 ± 6.8	0.057
**LV GLS [%]**	−14 ± 3.1	−15.7 ± 3.7	0.015
**Other Parameters**			
**Zva [mmHg/mL/m^2^]**	5.6 ± 1.7	4.8 ± 1.4	0.003
**LA area [cm^2^]**	24.1 ± 6.2	21.3 ± 4.9	0.009
**E/E′**	15.6 ± 7	10 ± 3.9	0.001

**Table 3:** Serum concentrations of the biomarkers. ATX—autotaxin; LPA—lysophosphatidic acid; GDF-15—growth differentiation factor-15; TM—thrombomodulin; Q1—first quartile; Q3—third quartile.

Biomarker	Patients with AS (N = 86)	Control Group (N= 63)	*p* Value
**ATX [ng/mL]**	112.07 ± 50.0	93.54 ± 27.2	0.003
**LPA [ng/mL]**	842.7 ± 508.1	928.3 ± 534.0	0.278
**GDF-15 [ng/mL]**	1.34 ± 1.4 (Q1: 0.64; Q3: 1.5)	0.71 ± 0.5 (Q1: 0.32; Q3: 0.94)	0.001
**TM [ng/mL]**	7.76 ± 4.0	6.3 ± 1.9	0.005

**Table 4:** Serum concentrations of the biomarkers in subgroups of AS patients. HG—high gradient; LG—low gradient; ATX—autotaxin; LPA—lysophosphatidic acid; GDF-15—growth differentiation factor-15; TM—thrombomodulin; Q1—first quartile; Q3—third quartile.

Biomarker	HG AS (N = 53)	LG AS (N = 24)	*p* Value
**ATX [ng/mL]**	111.89 ± 58.4	112.98 ± 34.07	0.55
**LPA [ng/mL]**	945.06 ± 550.9	756.86 ± 384.6	0.207
**GDF-15 [ng/mL]**	1.52 ± 1.7 (Q1: 0.81; Q3: 1.5)	1.11 ± 0.72 (Q1: 0.46; Q3: 1.7)	0.25
**TM [ng/mL]**	8.05 ± 4.9	7.49 ± 1.9	0.535

**Table 5:** The results of correlation analysis. r—Spearman’s rank correlation coefficient, *p*-significance. AVA—aortic valve area; Vmax—maximum velocity; Pmean—mean pressure gradient; ATX—autotaxin; LPA—lysophosphatidic acid; GDF-15—growth differentiation factor-15; TM—thrombomodulin; FMD—flow-mediated dilatation; LV GLS—left ventricular global longitudinal strain.

	AVA		Vmax		Pmean	
	r	*p*	r	*p*	r	*p*
**ATX**	−0.386	0.001	0.224	0.006	0.231	0.005
**LPA**	0.076	0.361	−0.012	0.888	0.004	0.957
**GDF-15**	−0.410	0.001	0.374	0.001	0.362	0.001
**TM**	−0.243	0.003	0.168	0.042	0.176	0.032
**FMD**	0.327	0.001	−0.310	0.001	−0.327	0.001
**LV GLS**	−0.286	0.001	0.222	0.008	0.250	0.002

**Table 6:** The results of regression analysis. R—coefficient of determination; ATX—autotaxin; AVA—aortic valve area; FMD—flow-mediated dilatation.

Dependent Variable	Independent Variable	R^2^	Regression Coefficient (β)	*p*-Value
ATX [ng/mL]	AVA [cm^2^]	0.145321	−20.93	0.011
ATX [ng/mL]	FMD [%]	0.145321	−13.42	0.012

**Table 7:** Predictive factors for total mortality and MACCE in 12-month follow-up. OR—odds ratio; AF—atrial fibrillation; NYHA—New York Heart Association; eGFR—estimated glomerular filtration rate; RVSP—right ventricular systolic pressure; GDF-15—growth differentiation factor-15.

Variable	OR (95% CI)	*p* Value
Predictive Factors for Mortality		
Age	1.0897 (1.0183–1.1662)	0.013
AF	4.4815 (1.0286–19.5246)	0.046
NYHA	2.6235 (1.2154–5.6629)	0.014
eGFR	0.9716 (0.9453–0.9986)	0.040
RVSP	1.0550 (1.0031–1.1097)	0.038
GDF-15	1.0006 (1.0000–1.0011)	0.036
Predictive Factors for MACCE		
Age	1.0507 (1.0050–1.0984)	0.029
Atherosclerotic plaque in ascending aorta	8.4507 (1.9119–37.3520)	0.005
eGFR	0.9630 (0.9414–0.9851)	0.001
GDF-15	1.0006 (1.0002–1.0011)	0.010

## Data Availability

The data presented in this study are available on request from the corresponding author due to privacy, intellectual property rights, and ethical reasons.

## Supplementary Materials

The following supporting information can be downloaded at: https://www.mdpi.com/article/10.3390/ijms26104873/s1.

## Abbreviations

The following abbreviations are used in this manuscript:

AF	atrial fibrillation
ALT	alanine aminotransferase
AS	aortic stenosis
ATX	autotaxin
AVA	aortic valve area
BMI	body mass index
CAD	coronary artery disease
CCS	Canadian Cardiovascular Society
CD	cluster of differentiation
COPD	chronic obstructive pulmonary disease
E/E′	ratio of early mitral inflow velocity to early diastolic mitral annular velocity
EDD	end-diastolic diameter
EDV	end-diastolic volume
EF	ejection fraction
ESD	end-systolic diameter
ESV	end-systolic volume
eGFR	estimated glomerular filtration rate
FMD	flow-mediated dilatation
GDF-15	growth differentiation factor-15
GLS	global longitudinal strain
HG	high gradient
HDL	high-density lipoprotein
IVS	interventricular septum thickness
LA area	left atrium area
LDL	low-density lipoprotein
LG	low gradient
LPA	lysophosphatidic acid
LV	left ventricular
MACCE	major adverse cardiovascular and cerebrovascular event
MCV	mean corpuscular volume
MPV	mean platelet volume
NYHA	New York Heart Association
PAD	peripheral artery disease
PDW	platelet distribution width
Pmax	maximum pressure gradient
Pmean	mean pressure gradient
PW	posterior wall thickness
Q1	first quartile
Q3	third quartile
SVi	stroke volume index
TM	thrombomodulin
TSH	thyroid-stimulating hormone
Vmax	maximum velocity
VICs	valvular interstitial cells
Zva	valvulo-arterial impedance
