# Supplementary material for: Biomarkers of Calcification, Endothelial Injury, and Platelet-Endothelial Interaction in Patients with Aortic Valve Stenosis
Source: Int J Mol Sci. 2025 May 19;26(10):4873. doi: 10.3390/ijms26104873 (PMC12112274; doi:10.3390/ijms26104873)
Supplement: Supplementary file 1 [file ijms-26-04873-s001.zip › ijms-3600924-supplementary.pdf]

Table S1. Laboratory characteristics. AS – aortic stenosis; MPV - mean platelet volume; PDW - platelet distribution width; ALT- alanine aminotransferase; eGFR - estimated glomerular filtration rate; TSH - thyroid-stimulating hormone; LDL - low-density lipoprotein; HDL - high-density lipoprotein. MCV- mean corpuscular volume; INR – international normalized ratio; APTT- activated partial thromboplastin time.

	Patients with AS (N=86)	Control group (N= 63)	P value
**Red blood cells [10^6/uL]**	4.2 ± 0.6	5.2 ± 5.0	0.001
**Hemoglobin [g/dL)**	12.8 ± 1.9	13.9 ± 1.7	0.001
**Hematocrit [%]**	37.9 ± 5.5	41.1 ± 4.1	0.001
**MCV [fL]**	88.5 ± 10.7	90.2 ± 3.9	0.346
**White blood cells [10^3/uL]**	7.2 ± 1.9	7.6 ± 2	0.351
**Neutrophils [10^3/uL]**	4.7 ± 1.7	4.9 ± 1.8	0.456
**Lymphocytes [10^3/uL]**	1.7 ± 0.6	1.9 ± 0.7	0.028
**Platelets [10^3/uL]**	204.8 ± 63	218.9 ± 66.9	0.137
**MPV [fL]**	10.7 ± 1	10.6 ± 1	0.238
**PDW [%]**	12.6 ± 2.2	12.4 ± 2.2	0.476
**ALT [IU/L]**	49.6 ± 238.8	31.4 ± 19.1	0.012
**Creatinine [mg/dL]**	1.2 ± 1.0	0.9 ± 0.2	0.001
**Sodium [mmol/L]**	138.9 ± 3.2	139.4 ± 2.1	0.832
**Potassium [mmol/L]**	4.2 ± 0.4	4.1 ± 0.4	0.011
**eGFR [mL/min]**	63.5 ± 22.3	75.5 ± 16	0.001
**TSH [μIU/mL]**	1.9 ± 0.9	1.9 ± 1.5	0.234
**Triglycerides [mg/dL]**	97.5 ± 32.5	118 ± 48.5	0.032
**Total cholesterol [mg/dL]**	156.5 ± 39.5	150.9 ± 45	0.215
**LDL [mg/dL]**	82.2 ± 33	78.9 ± 39.4	0.357
**HDL [mg/dL]**	54.8 ± 17.9	48.5 ± 12.4	0.039
**INR**	1.2 ± 0.6	1.05 ± 0.1	0.170
**APPT [sec.]**	31.5 ± 7.4	31 ±11.1	0.078

Table S2. Follow-up. AS – aortic stenosis; MACCE - major adverse cardiac and cerebrovascular events.

	**Patients with AS (N=86)**	**Control group (N= 63)**	**P value**
**MACCE**	18 (20.9%)	8 (12.7%)	0.191
**Bleeding (BARC>0)**	14 (16.3%)	8 (12.7%)	0.543
**Mortality**	14 (16.3%)	1(1.6%)	0.003
